# Glucan-rich polysaccharides from *Pleurotus sajor-caju* (Fr.) Singer prevents glucose intolerance, insulin resistance and inflammation in C57BL/6J mice fed a high-fat diet

**DOI:** 10.1186/1472-6882-12-261

**Published:** 2012-12-21

**Authors:** Gowri Kanagasabapathy, Umah Rani Kuppusamy, Sri Nurestri Abd Malek, Mahmood Ameen Abdulla, Kek-Heng Chua, Vikineswary Sabaratnam

**Affiliations:** 1 Institute of Biological Sciences, Faculty of Science, University of Malaya, 50603, Kuala Lumpur, Malaysia; 2 Department of Biomedical Science, Faculty of Medicine, University of Malaya, 50603, Kuala Lumpur, Malaysia; 3 Mushroom Research Centre, University of Malaya, 50603, Kuala Lumpur, Malaysia

**Keywords:** *Pleurotus sajor-caju*, Diabetes, Polysaccharides, C57BL/6J mice, High-fat diet

## Abstract

**Background:**

*Pleurotus sajor-caju* (*P. sajor-caju*) has been extremely useful in the prevention of diabetes mellitus due to its low fat and high soluble fiber content for thousands of years. Insulin resistance is a key component in the development of diabetes mellitus which is caused by inflammation. In this study, we aimed to investigate the *in vivo* efficacy of glucan-rich polysaccharide of *P. sajor-caju* (GE) against diabetes mellitus and inflammation in C57BL/6J mice fed a high-fat diet.

**Methods:**

Diabetes was induced in C57BL/6J mice by feeding a high-fat diet. The mice were randomly assigned to 7 groups (n=6 per group). The control groups in this study were ND (for normal diet) and HFD (for high-fat diet). The treated groups were ND240 (for normal diet) (240 mg/kg b.w) and HFD60, HFD120 and HFD240 (for high-fat), where the mice were administrated with three dosages of GE (60, 120, 240 mg GE/kg b.w respectively). Metformin (2 mg/kg b.w) served as positive control. The glucose tolerance test, glucose and insulin levels were measured at the end of 16 weeks. Expressions of genes for inflammatory markers, GLUT-4 and adiponectin in the adipose tissue of the mice were assessed. One-way ANOVA and Duncan’s multiple range tests (DMRT) were used to determine the significant differences between groups.

**Results:**

GE treated groups improved the glucose tolerance, attenuated hyperglycemia and hyperinsulinemia in the mice by up-regulating the adiponectin and GLUT-4 gene expressions. The mice in GE treated groups did not develop insulin resistance. GE also down-regulated the expression of inflammatory markers (IL-6, TNF-α, SAA2, CRP and MCP-1) via attenuation of nuclear transcription factors (NF-κB).

**Conclusion:**

Glucan-rich polysaccharide of *P. sajor-caju* can serve as a potential agent for prevention of glucose intolerance, insulin resistance and inflammation.

## Background

Diabetes is one of the leading non-communicable diseases affecting mankind [1]. It is estimated more than 284 million people are diabetic worldwide and this figure is predicted to double by 2030 [2]. Diabetes mellitus (DM) is a complicated metabolic disorder, characterised by high blood glucose level due to the inability of cells to utilise glucose appropriately. The etiology of type-1 diabetes is the absolute deficiency of insulin secretion, while type 2 diabetes (DM) is a combination of resistance to insulin action and impaired insulin secretion, which accounts for more than 90% of all diabetes cases [3]. Diabetes may lead to microvascular (blindness, renal failure and neuropathy) and macrovascular (stroke and myocardial infarction) complications [4]. It is also considered to be an important risk factor for the development of obesity, hyperinsulinemia, hypertension, dyslipidemia and atherosclerosis [5].

The current treatment for DM includes insulin and other oral hypoglycemic drugs such as sulphonylurea derivatives, biguanides, thiazolidinediones and alpha glucosidase inhibitors. However, these agents are known to have undesirable side effects such as high blood pressure, dry mouth, constipation, headache, valvular heart disease and obesity [6]. To date, natural products still play an important role as sources of medicine in preventing diabetes thus, the efforts to discover useful drug candidates to combat diabetic complications are going on relentlessly [7].

Mushrooms are well recognized for their medicinal properties and have been used in traditional medicine for centuries. The medicinal effects of mushrooms include antioxidant, antiviral, antibacterial, antifungal, anti-parasitic, detoxification, immunomodulatory, antitumor, radical scavenging, anti-inflammatory, cardiovascular, anti-hyperlipidemic or anti-hypercholesterolemic, hepatoprotective and anti-diabetic [8]. Edible mushrooms have been used to maintain health and increase longevity since ancient times [9]. In Malaysia, the widely cultivated ‘edible fungal food’ is the genus *Pleurotus*, commonly referred to as ‘oyster mushrooms’. Oyster mushrooms have been discovered to have definite nutritive and medicinal values and are most popular in countries such as India, China and Japan. Currently, *Pleurotus sajor-caju* is cultivated throughout the world. It contains good quality proteins and vitamins such as B_1_, B_2_, and C and has very little lipid or starch. This mushroom is claimed to be able to reduce the cholesterol level in blood [10] and prevent hyperlipidemia and this ability is attributed to its low fat and high soluble fiber content [11].

There are two distinct animal models of obesity; the first type is genetic obesity, as seen in rodent strains such as the Zucker fatty (*fa/fa*) rat and the leptin-deficient obese (*lep*^*ob*^*/lep*^*ob*^) mouse, which become obese under various experimental conditions. The second type of mouse model for obesity, C57BL/6J reflects the human condition more closely where the animals develop central adiposity, hyperinsulinemia and hyperglycemia as a result of a combination of genetic and environmental factors such as long term high-fat intake [12]. Hence in this study, the C57BL/6J mouse strain was selected to investigate the potential effects of glucan-rich polysaccharides of *P. sajor-caju* in mice fed a high fat-diet on metabolic changes pertaining to glucose homeostasis and associated inflammation. The activity was also compared with metformin (a widely used oral anti-diabetic drug).

## Methods

### Mushrooms samples

Fresh fruiting bodies of *Pleurotus sajor –caju* (10kg) were collected from a mushroom farm in Semenyih (Location - 3°21’19.20”N 101°14’36.35”E), Selangor Darul Ehsan, Malaysia.

### Isolation and purification of glucan-rich polysaccharide from hot-aqueous extract of *P. sajor-caju* (GE)

The isolation and purification of polysaccharide were based on the method described previously [13]. Fruiting bodies of *P. sajor-caju* was collected and washed with water. It was crushed and boiled in 500 ml of distilled water for 8 hours. The whole mixture was kept overnight at 4°C and then filtered through a linen cloth. The filtrate was centrifuged at 13000 × g for 45 minutes at 4°C. The supernatant was collected and precipitated in ethanol (1:5 [v/v]). It was kept overnight at 4°C and again centrifuged at 13000 × g for 45 minutes. The precipitated material (polysaccharide) was washed with ethanol four times and then freeze-dried. The freeze-dried material was dissolved in 30 ml of distilled water and dialyzed through dialysis tubing made up of cellulose membrane (Sigma-Aldrich, USA) against distilled water for 4 hours. This procedure removed low molecular weight materials. The aqueous solution was then collected from the dialysis bag and freeze-dried to yield crude polysaccharide (GE). The β-glucan level in the extract was estimated using a ß - glucan kit (specific for mushroom and yeast) purchased from Meganzyme International (Ireland).

### Animals

This study was conducted in conformity with the policies and procedures of the Animal Care and Use Committee of Faculty of Medicine, University of Malaya, with reference to the 8th edition: Guide for the Care and Use of Laboratory Animals by the Institute of Laboratory Animal Research, National Academy of Science, USA. The animal ethics approval was obtained from Animal Care and Use Committee of Faculty of Medicine, University of Malaya (IACUC, UM) (Approval number:ISB/14/07/2010/GK [R]). Female C57BL/6J (*ob/ob*) mice (7-weeks old) were purchased from BioLasco Laboratory, Taiwan. The animals were kept in stainless steel wire–mesh cages in a room maintained at 21°C and a standard condition of 12-hour light/dark cycle (light period: 8:00–20:00 hour). The animals were allowed free access to food and water, which were provided fresh every day.

### Experimental design

After one week of acclimatisation, the mice were randomly assigned (based on weight) into seven groups (n=6/group). Table 1 shows the type of diet and concentration of GE administrated for each group. The control groups in this study were ND (for normal diet) and HFD (for high-fat diet), where the mice were administrated with distilled water. The treated groups were ND240 (for normal diet) and HFD60, HFD120 and HFD240 (for high-fat), where the mice were administrated with different dosages of GE and metformin served as positive control. The composition of fat in normal diet was 5% of total energy whilst for the high-fat diet was 45 or 60% of fat. GE was administered thrice a week, via epi-gastric route using a feeding needle (size 20) to groups ND240, HFD60, HFD120 and HFD240 for 16 weeks. After 7 weeks of feeding with 45% of fat (TestDiet®, USA), the animal diet was substituted with 60% of fat (TestDiet®, USA) for groups HFD, HFD60, HFD120, HFD240 and HFDMET whilst for groups ND and ND240 the diet was not altered throughout the experiment.

**Table 1:** Type of diet and concentration of GE administrated to each group

**Type of diet**	**Groups**	**Treatment**
**Normal diet**	ND	Normal diet only + distilled H_2_O
	ND240	Normal diet + 240 mg/kg of body weight GE
	HFD	High-fat diet only + distilled H_2_O
	HFD60	High-fat diet + 60 mg/kg of body weight of GE
**High-fat diet**	HFD120	High-fat diet + 120 mg/kg of body weight of GE
	HFD240	High-fat diet + 240 mg/kg of body weight of GE
	HFDMET	High-fat diet + 2 mg/kg of body weight of metformin (anti-diabetic drug)

### Oral Glucose Tolerance Test (OGTT)

At the end of 16 weeks, the oral glucose test was carried out on the mice after subjecting to an overnight fast. Blood samples were obtained from a cut in the tail vein of the mice and blood glucose levels were determined using ACCU-CHEK® glucometer and ACCU-CHEK® Advantage test strips. Firstly, the fasting blood glucose levels were measured. Then, GE was administrated to the mice via epi-gastric route using a feeding needle and the blood glucose levels was measured again. Glucose (2 g/kg of body weight) was also administrated to the mice via epi-gastric route. Finally, the blood glucose levels were measured every 30 min for 2 hours (30, 60, 90 and 120 min). According to the protocol of the kit and Lee et al., [14] mice with fasting blood glucose level of 7.8 mmol/L (200 mg/dL) and above were considered hyperglycemic meanwhile mice with fasting blood glucose of below 3.9 mmol/L (70 mg/dL) was categorized as hypoglycemic.

### Sample collection and analytical methods

At the end of 16 weeks, the mice were anesthetized with ether after withholding food for 12 hours and were sacrificed by aortic exsanguination. Blood samples were collected in SST™ glass serum tubes with gold BD Hemogard™ closure (BD Vacutainer®, USA). Serum samples were separated after centrifugation at 2400 × g for 15 minutes. The samples were stored at −80°C for the estimation of insulin level. Adipose tissues were removed and stored in RNAlater® solution (Applied Biosystems, USA) and refrigerated at 4°C overnight before storing the samples at −80°C for RNA extraction.

### Insulin Sensitivity Test

The insulin sensitivity assay was done using Rat Insulin Enzyme Immunoassay kit (SPI-BIO Bertin pharma, France). The assay is based on the competition between unlabeled rat insulin and acethylcholinesterase (AChE) linked to rat insulin (tracer) for limited specific Guinea-Pig anti-rat insulin antiserum sites. The absorbance was read at 410 nm using a microplate reader (Bio-Tek Instruments Inc, USA). Rat insulin of known concentrations (0.08 to 10 ng/ml) was used as a standard for the estimation of insulin concentration and the results were expressed as ng/ml.

The index of insulin resistance was estimated by the homeostasis model assessment (HOMA) and was calculated using relationship between the blood glucose and insulin levels according to the following formula [15]:

(1) $$\mathrm{HOMA}-\mathrm{IR}=\frac{\mathrm{Insulin}\;\left(\mu \mathrm{UI}/L\right)\times \mathrm{Blood}\;\mathrm{glucose}\;\left(\mathrm{mmol}/L\right)}{22.5}$$

### Gene expression studies using Real Time – RT-PCR

The total RNA was isolated from the adipose tissue of all the mice groups as stipulated in Table 1 using Ambion- RNAqueous Micro® kit (Applied Biosystems, USA). The purity of recovered total RNA was estimated by calculating the ratio of absorbance reading of 260 nm and 280 nm. Purified RNA with a A_260_/A_280_ ratio between 1.8 - 2.0 was further used to synthesize complementary DNA (cDNA) by polymerase chain reaction (PCR) approach. The integrity of RNA was estimated using Agilent® 2100 Bioanalyzer (Applied Biosystem, USA). RNA samples with RIN value 8–10 were used in this study. High Capacity cDNA Reverse Transcription Kit (Applied Biosystem, USA) which contains all reagents needed for reverse transcription (RT) of total RNA to single-stranded cDNA was used in this study. Generally, 10 μl of RNA sample was mixed with 10 μl of High Capacity cDNA Reverse Transcription (RT buffer, dNTP mix, randoms primers, Multiscribe reverse™ transcriptase enzyme and nuclease free water). The mixture was then loaded into a thermal cycler (Eppendorf, USA) and PCR was carried out according to optimized thermal cycling conditions as recommended by the manufacturer. Table 2 shows the list of genes investigated in this study and the corresponding accession numbers. Endogenous control (also known as housekeeping genes) used in this study was eukaryotic 18S rRNA with FAM/MGB probe. All TaqMan® (Applied Biosystems, USA) probes used in this investigation were labeled with FAM™ reporter dye at the 5’ end and MGB quencher at the 3’ end. The quantification approach used was comparative C_T_ method, also known as 2 ^–ΔΔCt^ method [16].

**Table 2:** Genes investigated

**No**	**Gene name and abbreviation**	**Assay ID**	**Accession number**
**1**	Adiponectin	Mm 00456425_m1	NM_009605.4
**2**	Glucose transporter	Mm 00436615_m1	NM_009204.2
	(GLUT-4)		
**3**	Retinol binding protein 4	Mm 00803266_m1	XM_993476
	(RBP-4)		
**4**	Nuclear factor-κB	Mm 00482418_m1	NM_003998
	(NF-κB)		
**5**	Tumor necrosis factor-α	Mm 00443258_m1	NM_013693.2
	(TNF-α)		
**6**	Serum amyloid A 2	Mm 00656927_g1	NM_009117.3
	(SAA-2)		
**7**	Interleukin 6	Mm 00446190_m1	NM_031168.1
	(IL-6)		
**8**	Monocyte chemoattractant protein-1	Mm 00437433_m1	NM_011331.2
	(MCP-1)		
**9**	C-reactive proteins	Mm 00432680_g1	NM_007768.4
	(CRP)		

General abbreviation of genes selected for this study and corresponding assay ID and accession number was obtained from the Applied Biosystems website and NCBI database. Assay ID refers to the Applied Biosystems Gene Expression Assays inventoried kits with proprietary primer and TaqMan® probe mix. Assay ID with ‘Mm’ is referred to as ‘*Mus musculus’*. All Gene Expression Assay kits indicated are FAM/MGB probed.

### Statistical analysis

Data are shown as mean ± SD of triplicate assays. One-way analysis of variance was used to determine the significant differences between groups. Statistical significance was accepted at p < 0.05. Duncan’s multiple range tests (DMRT) was used to determine the significant differences between groups. SPSS Statistic software (version 17.0, IBM Corp., USA) was used for all statistical analyses. All figures were drawn using GraphPad Prism 5 (GraphPad Software Inc., California, USA).

## Results and discussion

### Weight and estimation of β-glucan concentration in GE

GE (12.31g) was obtained from 5500g of fresh *P. sajor-caju* fruiting bodies. This extract was then used to calculate the content of β-glucan by measuring the concentration of total glucan and α-glucan. The concentration of total glucan in GE was 85.95% (w/w) and the concentrations of α-glucan and β-glucan were 5.4% (w/w) and 80.55% (w/w) respectively. The percentage of β-glucan and α-glucan in dried *P. sajor-caju* were 1.5% and 0.01% (w/w) respectively. In this study, a detailed analysis of the sugar composition and lingkages was not attempted but Pramanik et al. [17], reported that the polysaccharides in *P.sajor-caju* consists of repeating unit of D-glucose, D-galactose, and D-mannose in a molar proportion of 1:1:1 with (1→3),(1→6)-β-glucans and (1→3)-α-glucans linkages.

### Effects of GE on glucose tolerance

**Figure 1:**
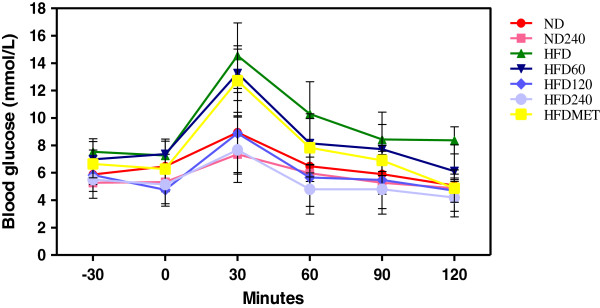
**Effects of GE on oral glucose tolerance (OGT) in C57BL/6J mice fed a high-fat diet or normal diet.** GE concentrations were 60, 120, 240 mg/kg/day. Metformin (MET) was used as positive control. Blood glucose was measured 30 minutes before glucose administration (−30 min) and every 30 minutes after glucose administration (0, 30, 60, 90, 120 min). Values expressed are means ± S.D of triplicate measurements (n=6 per group).

The glucose tolerance test was performed on the mice. Figure 1 shows the oral glucose tolerance test results of all the groups. Mice in HFD and HFD60 groups had elevated fasting blood glucose level and reduced glucose tolerance. In GE treated mice (ND240, HFD120 and HFD240), the glucose level in the first 30 minutes (after the initial glucose load) were lower than HFD while the glucose level at 120 minutes was within the normal range and the activity was similar to the metformin treated group (HFDMET). The blood glucose tolerance level in descending order was ND240 > HFD240 > ND > HFD120 > HFDMET > HFD60 > HFD.

### Effects of GE on blood glucose, serum insulin levels and insulin resistance index

Systemic inflammation has been recognized as a key link between insulin resistance and diabetes [18]. Insulin resistance is characterized by increased expression of pro-inflammatory cytokines, macrophage infiltration into white adipose tissue and an impaired response to insulin in the main insulin target tissues.

**Figure 2:**
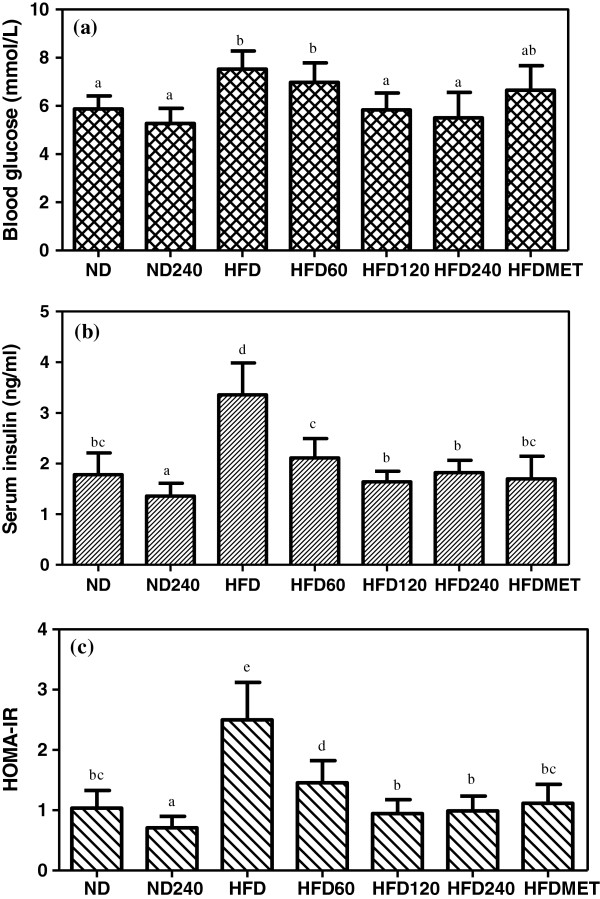
**Effects of GE on (a) fasting blood glucose concentrations, (b) serum insulin levels and (c) HOMA-IR value of C57BL/6J mice fed a high-fat diet or normal diet.** GE concentrations were 60, 120, 240 mg/kg/day. Metformin (MET) was used as positive control. Values expressed are means ± S.D of triplicate measurements (n=6 per group). Means with different alphabets in different bar (a-e) are significantly different (p<0.05).

Figure 2 (a-c) shows the effect of GE and metformin on blood glucose levels, serum insulin concentration and HOMA-IR value in mice fed a high-fat diet or normal diet. The fasting blood glucose in descending order was HFD > HFD60 > HFDMET > HFD > ND > HFD120 > HFD240 > ND240. A moderate but significant hyperglycemia developed in HFD group. Severe hyperglycemia and hyperinsulinemia in C57BL/6J mice are only known to develop after 24 weeks of feeding with high-fat diet [19]. GE administrated mice (HFD120 and HFD240) showed a significantly lower fasting blood glucose levels compared to the mice in HFD group. Nevertheless, there were no significant differences observed in the normal diet groups (ND and ND240). The serum insulin level was significantly elevated in HFD group and was 88.8% higher than the ND group which indicates hyperinsulinemia. However, GE treated groups and metformin treated group showed a significant lower serum insulin concentration compared to HFD group.

Homeostatic model assessment values for insulin resistance (HOMA-IR) were calculated using the formula stated above. The HOMA-IR indices for the HFD group showed an increase of 150% compared to ND group. In spite of feeding a high-fat diet, the insulin resistance indices for HFD60, HFD120 and HFD240 groups were significantly reduced by 44%, 62.4% and 60.8% respectively whilst in HFDMET group, the insulin resistance index was significantly reduced by 56% compared to HFD group. Amelioration of insulin resistance in HFD240 treated group was comparable to the metformin treated group. Thus it is pertinent to suggest that GE was able to prevent hyperglycemia by preventing insulin resistance in the mice fed a high-fat diet.

Reactive oxygen species (ROS) production is one of the many factors that have been suggested to play a role in the development of insulin resistance [19,20]. Nagata et al. [21] have demonstrated that up-regulation of genes is responsible for ROS production occurs in both the liver and adipose tissue before the onset of insulin resistance in mice fed a high fat-diet.

### Effects of GE on the expression of adipokines and inflammatory markers in adipose tissue

Adipose tissue is actively involved in sensing the nutritional and metabolic status of the organism through several signalling pathways and the energy metabolism is regulated by the secretion of molecules in response to these cues [22]. It is a complex and active secretory organ that both sends and receives signals that modulate energy expenditure, appetite, insulin sensitivity, endocrine function, inflammation and immunity [23]. Thus, this part of the study, attempts to increase the understanding of how GE possibly regulates the underlying mechanism responsible for hypoglycemia, amelioration of insulin resistance and the associated inflammation. Table 3 shows the expression of adipocyte derived cytokines (adipokines).

**Table 3:** Effects of GE on the expression of adipokines in adipose tissue

**Genes investigated**	**ND240**	**HFD60**	**HFD120**	**HFD240**	**HFDMET**
**GLUT-4**	1.49±0.24	1.78±0.3b^d^	2.41±0.42^b^	2.05±0.32^a^	1.13±0.13^c^
**Adiponectin**	1.56±0.15	0.81±0.37^c^	1.13±0.62^b^	1.68±0.30^a^	1.66±0.24^a^
**RBP-4**	0.10±0.13	−1.20±0.16^b^	−1.30±0.44^b^	−1.63±0.33^a^	−1.50±0.17^a^

Results are expressed as –fold variation over the appropriate control groups; ND240 indicate fold increase over ND, normal diet control group and HFD60, HFD120, HFD240 and HFDMET indicate fold increase over HFD, high-fat diet control group. Fold variations less than one were expressed as negative numbers (e.g., a –fold variation of 0.50 is expressed as −2.00). Values expressed are means ± S.D of triplicate measurements. Statistical significance was calculated based on the mean ΔCT values by DMRT for only mice fed with high-fat diet with or without GE. For same gene with different treatment groups, means in the different rows with different alphabets (a-d) were significantly different (p<0.05).

After 15 weeks of feeding a high-fat diet, HFD group mice developed hyperglycemia and had reduced glucose tolerance (Figure 1). This could be attributed to the reduced expression of adiponectin. Meanwhile, GE treated groups (HFD120 and HFD240) had increased adiponectin expression and this may explain the better glucose tolerance observed in these groups. Adiponectin is considered to be an anti-diabetic and anti-atherogenic hormone, but conflicting results on its levels in obese and diabetic patients and animal models have been shown in previous studies [24]. Some of them demonstrated declined circulating levels of adiponectin in high-fat diet fed rats [25] and mice [26] while others did not observe any changes [27,28]. A possible explanation for such difference is that the expression of adiponectin, could be altered by multiple factors including both genetic and environmental factors, as well as resting and fasting stages [29]. Meanwhile, GLUT-4 gene, the insulin-responsive glucose transporter, plays a major role in glucose transport. The maintenance of insulin sensitivity in adipose tissue and the development of insulin resistance (IR) is a result of impaired GLUT-4 signalling pathway [30]. In this study, the mice in GE treated groups, showed better glucose tolerance than the mice in HFD group, and the improvement in glucose tolerance was also reflected by the increased GLUT-4 expression in these groups.

On the contrary, RBP-4 has been reported as a factor that is derived from adipose tissue that can cause insulin resistance [31]. The mechanism of RBP-4 activation is still unclear but several reports have shown that increased expression of RBP-4 was found in adipose tissue of mice with adipocyte-specific ablation of GLUT-4 [32]. Increased plasma RBP-4 levels in obese children correlated not only with insulin resistance but also with inflammatory factors [33]. This concurs with the findings in this study that, GE treated groups (HFD60, HFD120 and HFD240) had decreased expression of RPB-4 compared to HFD group. In HFDMET group, the regulation of these adipokines was similar to the GE treated groups.

The expressions of inflammatory markers (IL-6, CRP, MCP-1, SAA-2, NF-κB and TNF-α) were also assessed in the adipose tissue as shown in Table 4. The link between diabetes mellitus, insulin resistance and inflammation are well known [34] and epidemiological evidence has confirmed these findings by showing an increase of additional acute phase reactants in diabetic subjects, including TNF-α, IL-6 and CRP [35]. Glucose and fat intake have both been shown to induce inflammation, possibly due to increase of oxidative stress [36].

**Table 4:** Effects of GE on the expression of inflammatory markers in adipose tissue

**Genes investigated**	**ND240**	**HFD60**	**HFD120**	**HFD240**	**HFDMET**
**IL-6**	−1.17±0.67	−1.38±0.41^c^	−1.11±0.20^d^	−3.27±0.92^b^	−1.35±0.03^c^
**CRP**	−2.10±0.60	0.10±0.78^a^	−4.00±0.21^b^	−3.40±0.87^b^	−2.30±0.12^c^
**MCP-1**	−1.63±0.45	1.09±0.14^b^	−2.33±0.24^c^	−1.33±0.69^d^	−1.20±1.10^d^
**SAA-2**	−1.55±0.66	−5.53±0.47^b^	−5.39±0.54^b^	−5.67±0.95^b^	−3.82±0.77^c^
**NF-κB**	−1.11±0.37	−2.30±0.29^b^	−2.90±0.92^c^	−2.30±0.30^b^	−2.45±1.13^b^
**TNF-α**	−3.46±0.09	1.16±0.21^b^	−4.50±0.43^c^	−4.11±0.37^c^	−1.79±1.06^d^

Results are expressed as –fold variation over the appropriate control groups; ND240 indicate fold increase over ND, normal diet control group and HFD60, HFD120, HFD240 and HFDMET indicate fold increase over HFD, high-fat diet control group. Fold variations less than one were expressed as negative numbers (e.g., a –fold variation of 0.50 is expressed as −2.00). Values expressed are means ± S.D of triplicate measurements. Statistical significance was calculated based on the mean ΔCT values by DMRT for only mice fed with high-fat diet with or without GE. For same gene with different treatment groups, means in the different rows with different alphabets (a-d) were significantly different (p<0.05).

Nuclear factor-κB (NF-κB) controls the regulation of the genes that encode proteins involved in immune and inflammatory responses (i.e., cytokines, chemokines, growth factor immune receptors, cellular ligands, and adhesion molecules) [37]. Diabetes causes reduction in the production of anti-inflammatory adipokines (like adiponectin) and increase in the production of pro-inflammatory cytokines (like IL-6, CRP and SAA-2). This, in turn, leads to the MCP-1 facilitated infiltration of monocytes and macrophages into the adipose tissue [38]. The inflamed state leads to the release of cytokines into adipose tissues that are not normally secreted by the adipose cells. A key cytokine that is released by macrophages but not by differentiated adipocyte, is TNF-α, the key regulator of inflammation which is strongly correlated to the level of insulin resistance in obese and diabetic animal models [39]. Studies have demonstrated that, TNF-α along with IL-6 not only inhibit adiponectin (anti-inflammatory adipokines) expression and impair insulin sensitivity of other tissues but also interfere with adipocyte metabolism at numerous sites including transcriptional regulation, glucose and fatty acid metabolism [40]. The pro-inflammatory cytokine IL-6 was among the first to be implicated as a predictor of insulin resistance. Finally, these pro-inflammatory cytokines (IL-6 and TNF-α) mediate distant inflammatory effects, including activation of CRP and SAA-2 [41]. The SAA proteins are derived from distinct genes; human express SAA-1, SAA-2, SAA-3 and SAA-4. In this study, only SAA-2 protein was studied because SAA-2 expression increases dramatically during acute inflammatory responses and adipocytes have been suggested to be a major contributor of SAA-2 [23]. Meanwhile, CRP is an acute phase reactant. Circulating concentrations of SAA-2 and CRP are also increased in individuals with impaired glucose tolerance and are considered to be a marker for insulin resistance [42].

The HFD group had higher expression of the pro-inflammatory markers compared to the GE treated groups and metformin treated group. ROS and endoplasmic reticulum (ER) stress have been reported to be increased by adiposity and consumption of high-fat diet may activate NF-κB signalling cascade in the adipose tissue [23]. When NF-κB signaling cascade is activated, increased MCP-1 results in a dramatic elevation of macrophage infiltration, promoting the downstream secretion of pro-inflammatory markers namely, TNF-α and IL-6 and finally this increases the expression of CRP and SAA-2. The expression of these pro-inflammatory markers in HFD, are known to initiate and amplify insulin resistance in the adipose tissue as observed in this study. However, the NF-κB activity in GE and metformin treated groups, was significantly down-regulated compared to the HFD group. Thus, the expression of TNF-α and IL-6 also decreased in these groups. Similarly, the expressions of SAA-2 and CRP were also down-regulated in GE and metformin treated groups compared to HFD group. Besides that, the local enhancement in adiponectin expression induced by GE might also be partly responsible for the reduced inflammatory-cytokine expression levels in these groups [39]. Similar findings have been reported on adipose tissue treated with various natural substances such as grape seed procyanidins [38] and tocotrienol [37].

## Conclusion

Polysaccharide of *P. sajor-caju* which is rich in β-glucans (GE) (240mg/kg of body weight) prevented the occurrence of glucose intolerance, hyperglycemia and hyperinsulinemia/insulin resistance in C57BL/6J mice fed a high-fat diet by up-regulating the expression of GLUT-4 and adiponectin genes and down-regulating the expression of NF-κB. The properties displayed by GE were comparable to metformin (positive control). GE could serve as a potential candidate for prevention of glucose intolerance, insulin resistance and inflammation.

## Competing interests

The authors declare that they have no competing interests.

## Authors’ contribution

GK was responsible for the methodology execution, data analysis and writing the manuscript. URK was responsible for providing grants, conception of ideas, data interpretation and revising the manuscript, SNAM was responsible for the isolation of polysaccharide from the mushroom, MAA was responsible for the animal experiment, CKH was responsible for the gene expression studies and VS was responsible for conception of idea and revising the manuscript. All authors read and approved the final manuscript.

## Pre-publication history

The pre-publication history for this paper can be accessed here:

http://www.biomedcentral.com/1472-6882/12/261/prepub
